# A Deep Learning On-Board Health Monitoring Method for Landing Gear Shock-Absorbing Systems

**DOI:** 10.3390/s25092767

**Published:** 2025-04-27

**Authors:** Chunsheng Li, Wang Chen, Wenfeng Qin

**Affiliations:** Department of Aircraft Engineering, Civil Aviation Flight University of China, Deyang 618307, China; cwwyyx0201@163.com (W.C.); qwfgrh@126.com (W.Q.)

**Keywords:** deep learning, health monitoring, landing gear, shock-absorbing system, general aviation aircraft, multi-body dynamic simulation

## Abstract

**Highlights:**

This paper proposed a new health monitoring method for landing gear shock-absorbing systems based on the dynamic responses of landing gears during aircraft landing phase. The proposed sensor network consists of vertical fuselage and wheel accelerometers, and a deep learning method LDGNet is proposed to conduct health monitoring for three typical shock absorber faults. The proposed health monitoring method is proved to be with good performance for all considered fault conditions.

**Abstract:**

This paper proposed a deep learning on-board health monitoring method for landing gear shock-absorbing systems based on dynamic responses during landing. A deep learning model is developed to conduct health monitoring for faults in shock absorbers. A certain general aviation aircraft is focused on in this paper, and a multi-body dynamic model of the nose landing gear is developed to simulate dynamic responses during landing under various health states and various landing conditions for developing a database for the proposed LDGNet. The simulated database is used to conduct model training and to test the performance of the proposed method. The feasibility and effectiveness of the proposed method are verified.

## 1. Introduction

Shock-absorbing systems are crucially important to ensure passenger comfort and protect cargo and fuselage structure during takeoff, landing and taxiing. Oleo-pneumatic shock absorbers are widely applied to offer stiffness and damping for absorbing impacts during landing. Faults and failures would occur on oleo-pneumatic shock absorbers under impacts during aircraft running operations, such as variations in oleo state and pneumatic leakages. Based on the statistics of a certain airline, four pneumatic leakage faults would occur in landing gear shock-absorbing systems among 100 aircraft in a year [1].

Current inspections of landing gear shock-absorbing systems are usually conducted manually based on the Aircraft Maintenance Manual (AMM) after running operations, and detailed inspections would require jacking up the aircraft in the hangar. Such calendar-based manual inspections would involve a high labor cost and could not offer fault predictions. On-board health monitoring systems for landing gear are promising due to the advantages of accuracy, low labor costs, real-time health monitoring, etc. The practical application of such system in aviation industry would face many problems, especially for general aviation aircraft. The landing gears of some general aviation aircraft are designed as simple structures, and are rarely equipped with sensors for health monitoring. Mounting additional sensor networks on landing gears would inevitably have negative effects on landing gear structure, and the application of such sensor networks is difficult to meet airworthiness requirements. Previously, the author of this paper conducted an overview of on-board health monitoring methods in the railway industry [2]. And based on the relationships between faults in the suspension system and dynamic system responses, an on-board health monitoring method was proposed for vehicle suspension systems with accelerometers mounted only on a car body [3,4]. Hence, it is desirable to explore on-board health monitoring methods for landing gear methods with limited sensors to pass airworthiness.

As a new generation of artificial intelligence technology, deep learning is widely applied in many industries [5]. Deep learning could automatically extract features from massive high-dimensional data through superimposed multi-layer neural networks and nonlinear transformations, and has the capabilities of self-learning, automatic updating and self-adaptation. Nowadays, deep learning is being widely used in natural language processing [6,7], image recognition [8,9], automatic driving [10,11] and target detection [12,13,14,15,16,17,18], etc.

Deep-learning is widely applied to conduct health monitoring and fault diagnosis for mechanical systems based on dynamic responses. A review was made for existing deep learning fault detection diagnosis methods of rotating machinery [15]. Recent deep learning methods have been divided into five main categories: deep belief networks (DBN), recurrent neural networks (RNN), autoencoders (AE), generative adversarial networks (GAN) and convolutional neural networks (CNN). Basic principles and applications of considered methods were made, and main problems of the considered methods were summarized. As a powerful tool for feature extraction, CNN is widely applied for machine fault diagnosis. CNN-based fault detection methods were reviewed by introducing the theoretical foundations, architectural variations and applications of CNNs, and discussing advancements, limitations and potential challenges [16]. The light neural network (LiNet) was proposed for rotating machines, and a CNN was applied to extract features from raw vibration signals [17]. The proposed LiNet was proven to have nearly 100% accuracy. A semi-supervised gear pitting fault diagnosis method was proposed based on deep sparse autoencoder and data transfer which has effective small sample enhancement capability and achieves more than 99% gear pitting fault diagnosis accuracy [18]. In addition to the above methods based on a single deep learning model, some scholars have proposed a method that integrates multiple deep learning models to diagnose rotating machinery faults. Ref. [19] proposed a framework combining the spatiotemporal pattern network (STPN) and CNN, which could adaptively extract deep features from spatiotemporal domain. A deep learning health-monitoring method was proposed to conduct health monitoring and fault diagnosis for wheel out-of-round of high-speed trains [20]. A multi-body dynamic model was developed to build a training database with simulated axlebox accelerations. The proposed method consisted of two one-dimensional convolutional neural networks (1DCNNs) and a 2DCNN to process features of time domain, frequency domain and time–frequency domain information of axlebox accelerations correspondingly, and all features were merged with FCNN and 3DCNN units to conduct fault detection. A CNN intelligent diagnosis method was proposed for bearing incipient faint faults [21]. After noise was reduced by the adaptive stochastic resonance, the position of the signal peak and the correlation coefficient between the divided signals were converted into a 2D image. A CNN was applied to conduct fault detection via extracting features from the 2D image. A method based on deep neural networks (DNN) was proposed to conduct automatic fault diagnosis of rolling bearings [22]. The effects of different sequence segmentation time lengths on fault diagnosis results were discussed in detail, and it was proved that the increase in sequence segmentation time would improve the accuracy of fault diagnosis. A novel convolutional neural network with a one-dimensional structure (ODCNN) was proposed to conduct automatic fault diagnosis of rolling bearings, which was proved with better performance compared with LeNet-5, AlexNet [23]. A hybrid deep learning method was proposed for bearing fault detection, based on CNN and gcForest [24]. In the proposed method. Continuous wavelet transform (CWT) was applied to convert bearing vibration signals into time–frequency images, CNN was applied to extract fault features, and gcForest was applied as a classifier. Refs. [25,26] both proposed the combination of CNN and long short-time memory (LSTM), which not only improved the efficiency of feature extraction and reduced the number of parameters, but also adapted to changing operating conditions. A CNN-LSTM-based fault detection method was proposed for wind turbines [27]. CNN was applied to extract dynamic changes in the acquired data and LSTM was enhanced by the attention mechanism to increase the accuracy of the proposed method.

Deep learning is also widely applied in the aviation industry [28]. A lightweight magnetic convolutional neural network (LMagNet) model was proposed to conduct hidden corrosion detection in aircraft structures [29]. To solve the problem of online monitoring of aero-engine rolling bearings, Ref. [30] proposed a hypersphere distance discrimination algorithm, which has the advantages of low computational complexity and no parameters need to be adjusted. The algorithm was applied to fault detection and degradation performance evaluation of aero-engine rolling bearings and achieved good results. Ref. [31] proposed a fault diagnosis method for aero-engine inter-shaft bearing based on rotor and cartridge vibration signals. Three typical deep learning methods were compared including CNN, LSTM and Transformer. Ref. [32] proposed a fault diagnosis method of aero-engine bearing based on the probability density information of rotor displacement (PIRD). The proposed method was proven with fast and accurate detection, combining 1DCNN end-to-end fault diagnosis advantages and reduced data redundancy by PIRD. Considering that insufficient aero-engine fault signals would not meet the requirement of deep learning model for big data, Ref. [33] proposed a domain-adversarial neural network (DANN) areo-engine rotor fault diagnosis method based on time–frequency analysis. Ref. [34] proposed an intelligent diagnosis of aero-engine rolling bearing faults based on deep transfer learning combining CNN and transfer learning.

This paper proposes an on-board health monitoring method for landing gear shock-absorbing systems based on analyzing signals of fuselage and wheel dynamic responses. The nose landing gear of a certain general aviation aircraft is focused on in this paper. The main work of this paper is summarized as follows:

(1) Modeling and simulations: a multi-body dynamic model is developed for the nose landing gear to simulate dynamic responses. The developed landing gear dynamic model consists of fuselage, engine frame, strut, shock absorber, fork, rim and tire. And the shock absorber is modeled in detail including damping force, air spring force, friction force and structural limiting force. Simulation results are compared with data from real flight to conduct model verifications. Simulations are conducted considering various health states of shock absorbers under various landing conditions to explore how faults in shock absorbers influence landing gear systems.
(2) Sensor network design: a sensor network is designed on fault effect evaluation. The proposed sensor network only consists of fuselage and wheel accelerometers to acquire vertical accelerations of fuselage and wheel during landing phase.
(3) Database: a virtual sensor network is applied on the developed nose landing gear model to acquire responses. A database is developed with sensor-acquired signals under various health states of shock absorbers at various landing conditions for training the proposed health monitoring method and evaluating its performance.
(4) Health monitoring method: a deep learning model LDGNet is proposed to conduct health monitoring for the landing gear shock-absorbing system. The proposed LDGNet mainly consists of CNN and LSTM to extract features in the time domain and the spatial domain. The developed database is applied to train LDGNet and conduct accuracy tests.

## 2. Problem Statement and Suggested Solutions

### 2.1. Sensor Network

A sensor network for monitoring component states is difficult to apply for landing gear due to various reasons. Firstly, the performance of landing gear shock-absorbing systems is affected by many factors, including oleo state, gas state, internal structural state, etc., which means that various sensor types are required by the sensor network. It would increase the cost of the designed system. Secondly, mounting sensors on landing gear would inevitably affect landing gear structure and present a difficulty in meeting airworthiness requirements. For example, shock absorbers need to be drilled to install additional sensors (e.g., pressure sensor) for conducting gas state monitoring, and structures need be drilled to install wires from the sensors. For the general aircraft considered in this paper, sensor wires would inevitably be exposed.

In this paper, a sensor network is designed to conduct health monitoring for landing gear shock-absorbing systems, via acquiring dynamic responses from the landing gear during the landing phase. Accelerometers are selected due to the fact that faults in shock-absorbing systems would deteriorate the dynamic responses of landing gear and its advantages including good performance, robustness, low cost, etc. Accelerometers are mounted on fuselage and wheel to acquire vertical accelerations during landing. The designed sensor network has a low cost and is more likely to pass airworthiness [35].

### 2.2. Health Monitoring Method

Implementing an on-board health monitoring system for landing gear would face various difficulties. Faults occurred in landing gear are decided by various aspects, including aircraft state (e.g., design, manufacture), pilot control, environment, maintenance, etc. Currently, it is difficult to describe the fault trigger and fault evolution mechanism. Deep learning methods could offer health monitoring with being trained by databases, instead of fault trigger and fault evolution mechanisms. For training an on-board health monitor method for landing gear, the database requires signals under all possible fault conditions and running conditions. It is difficult to acquire signals under fault conditions via practical aircraft flights. Mounting sensors on landing gear is difficult to pass airworthiness requirements, and flights with faults in the landing gear would increase the risk of accidents.

In this paper, a deep learning method LDGNet is proposed with the sensor network suggested in Section 2.1. A dynamic model of nose landing gear is developed for dynamic responses for the general aviation aircraft considered in this paper. Accuracy of the developed model is analyzed via comparing the simulation results with data from practical flights. Three common faults in shock-absorbing systems are considered in this paper, including damping orifice area (*A_S_*) faults, initial gas pressure (*P*_0_) faults and initial gas volume (*V*_0_) faults, and three typical landing conditions are considered, including soft, normal and heavy landing. For developing the training database, virtual sensors are mounted on the fuselage and wheel of landing gear model to acquire simulated dynamic signals under all considered conditions. The proposed LDGNet is trained by 80% of the database, and the performance of the LDGNet is tested by the remaining 20% of the database.

## 3. Nose Landing Gear Modeling

In this section, a multi-body dynamic model of nose landing gear is developed for simulating dynamic responses under various conditions.

### 3.1. Dynamic Modeling for Nose Landing Gear

**Figure 1:**
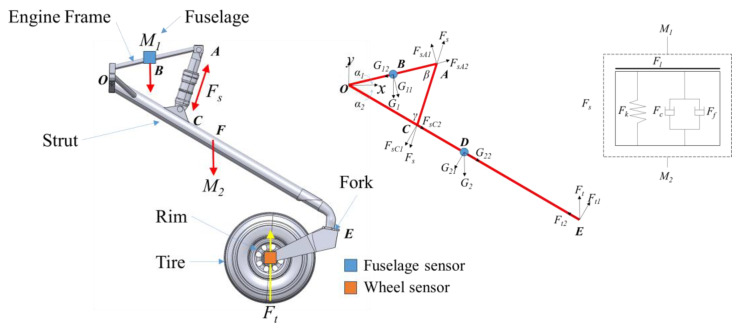
Nose landing gear of a certain general aviation aircraft.

As shown in Figure 1, the nose landing gear of the aimed general aviation aircraft mainly consists of the fuselage body, wheel, strut and shock absorber. Based on the force analysis shown in Figure 1, the nose landing gear is divided into damped mass *M*_1_ and un-damped mass *M*_2_. *M*_1_ mainly includes the fuselage weight, engine and engine frame. *M*_2_ mainly includes the wheel, strut, etc. In this paper, both *M*_1_ and *M*_2_ are modeled as rigid bodies connected with joints and a shock absorber. As shown in Figure 1, the mass center of *M*_1_ is fixed in the horizontal geometric center of the engine frame *B*. Forces on *M*_1_ mainly includes its own gravity *G*_1_ and axial force of shock absorber *F_s_*. For undamped mass *M*_2_, strut, fork and wheel are modeled as rigid body individually. Fork and wheel are assembled with joint of pitch rotation, fork and strut are assembled with joint of yaw rotation. Forces on *M*_2_ mainly include the gravity of the strut, fork and wheel of landing gear, the axial force of shock absorber *F_s_* and the impact on tire *F_t_*. The joints and degree of freedom (DOF) of the nose landing gear model are listed in Table 1.

According to load and joint analyses, dynamics equations for nose landing gear are shown as follows [36,37,38,39]:

(1) $$\left\{\begin{array}{l} M_{1}{\ddot{y}}_{1}L_{OB}\cos\alpha_{1}=M_{1}gL_{OB}\cos\alpha_{1}-F_{s}L_{OA}\cos\beta \\ M_{2}{{\ddot{\alpha }}_{2}}^{2}L_{OF}=M_{2}gL_{OD}\sin\gamma +F_{s}L_{OC}\sin\gamma -F_{t}L_{OE}\cos\alpha_{2}\\ L_{AC}(t)=\sqrt{L_{OA}^{2}+L_{OB}^{2}-2L_{OA}L_{OB}\cos(\alpha_{1}+\alpha_{2}(t))}\\ \frac{L_{AC}(t)}{\sin(\alpha_{1}+\alpha_{2}(t))}=\frac{L_{OA}}{\sin\gamma (t)}=\frac{L_{OC}}{\sin\beta (t)} \end{array}\right.$$

where *L_OA_*, *L_OB_*, *L_OC_*, *L_OD_* and *L_OE_* are distance between each force point and the rotation center *o*, respectively, *L_AC_* is the length of the shock absorber in real time, *α*_1_ is the pitching angle of the engine frame, *α*_2_ is the pitching angle of the strut, *β* is the angle between the shock absorber and engine frame and *γ* is the angle between the shock absorber and the strut.

Oleo-pneumatic shock absorbers offer gas spring stiffness and oil damping to take over vertical energy dissipation during landing. As shown in Figure 1, the axial force of the shock absorber *F_s_* mainly consists of gas spring force *F_k_*, oleo damping force *F_c_*, internal friction force *F_f_* and structural limiting force *F_L_*.

(2) $$\left\{\begin{array}{l} F_{s}(t)=F_{k}(t)+F_{c}(t)+F_{f}(t)+F_{l}(t)\\ F_{k}(t)=A_{0}\left[P_{0}{\left(\frac{V_{0}}{V_{0}-A_{0}L(t)}\right)}^{\alpha }-P_{atm}\right]\\ F_{c}(t)=\xi \frac{\rho {A_{1}}^{3}}{2{A_{s}}^{2}}\cdot \left|V_{c}(t)\right|V_{c}(t)\\ F_{f}(t)=k_{m}F{\left(t\right)}_{c}=\mu_{p}\frac{4h_{p}}{D}F{\left(t\right)}_{c}\\ F_{t}(t)=(1+C_{T}{\dot{y}}_{t}(t))f(y_{t}(t)) \end{array}\right.$$

where *A*_0_ is effective air pressure, which is mainly decided by the inner diameter of the piston rod, *P*_0_ is initial gas pressure, *V*_0_ is initial gas volume, *P_atm_* is local atmospheric pressure, *α* is gas variable coefficient. In this paper, the gas variable coefficient *α* is taken as 1.1 for considering the bubbles produced when the oil enters the air cavity and heat exchange with the air. *ρ* is oil density, *ζ* is the fluid damping coefficient of oil friction loss, *A*_1_ is oil pressure area and related to the inner diameter of the shock absorber outer cylinder, and *A_S_* is the damping orifice area. In this paper, no. 41 aviation hydraulic oil is chosen, and oil density *ρ* is taken as 873 kg/m^3^. In this paper, fluid damping coefficient of oil friction loss *ζ* is taken as 2. During the damping process of oleo-pneumatic shock absorber, the internal pressure of shock absorber and the bending of shock absorber pillar would cause the internal friction in the shock absorber. *k_m_* is the equivalent friction coefficient of the gland seal and related to seal thickness *h_p_*, the diameter of the piston rod *D* and the friction coefficient *μ_p_* between the seal and piston rod. *k_l_* is the axial tensile stiffness of the shock absorber. Structural limiting force *F_L_* would only work under abnormal landing conditions, such as heavy landing, gas or oil leakage in shock absorbers. *L_smax_* is the maximum travel of the shock absorber; in this paper, *L_smax_* is taken as 60 mm.

In the dynamic model of nose landing gear, the radial force generated by the contact between the wheel tire and ground is considered as spring-damping force, and the radial force of the wheel tire is calculated as follows:

(3) $$F_{t}(t)=(1+C_{T}{\dot{y}}_{t}(t))f(y_{t}(t))$$

where *C_T_* is the vertical damping of tire, *y_t_*(*t*) is the tire compression and *f*(*y_t_*(*t*)) is the static pressure curve of the tire.

In this paper, dynamic responses during practical aircraft landing are simulated as the free drop of the developed nose landing gear model at corresponding altitudes. In the simulations, the weight of *M*_1_ is 136 kg based on conversion. The initial rolling angle of the nose landing gear is 0°, the wheel and fork could rotate freely and the initial rotation speed of wheel is 0 km/h. A general horizontal road surface is selected for the drop simulation, collision force is added between the wheel and the ground, and the radial stiffness of the wheel tire is 1.9 × 10^5^ N/m. The simulation time is 6.0 and the sampling frequency is 1000 Hz.

**Figure 2:**
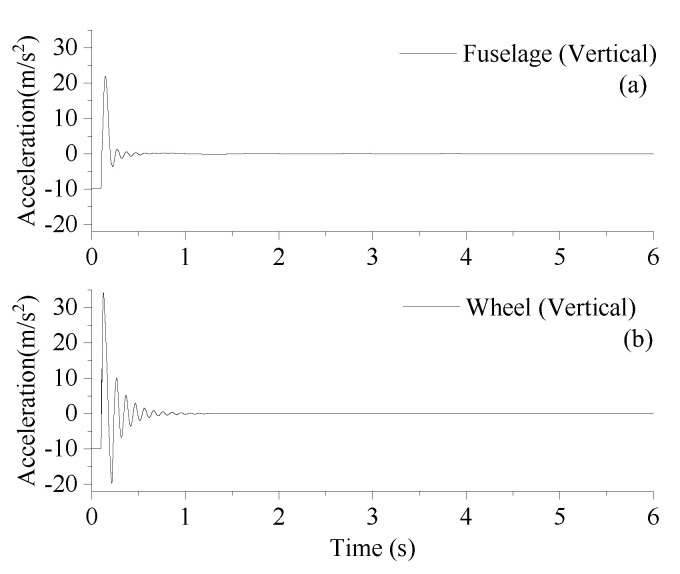
Vertical accelerations of (**a**) fuselage and (**b**) wheel under vertical landing speed of 1.0 m/s.

A normal landing condition (vertical landing speed of 1.0 m/s) is considered for the developed model by setting the initial altitude of *M*_1_ mass center as 0.963 m. Figure 2 shows vertical accelerations of the fuselage (a) and wheel (b) during the simulated landing phase at the vertical landing speed of 1.0 m/h. As shown in Figure 2, during the period from 0.0 s to 0.121 s, the nose landing gear is in free fall at gravitational acceleration, and the grounding speed is 1.0 m/s. From 0.121 s to 0.126 s, the shock absorber is compressed to the maximum travel and the wheel acceleration reaches the maximum peak. From 0.126 s to 0.214, the shock absorber stretches and the wheel acceleration reaches the negative maximum peak. After 0.598 s, the amplitude of vertical fuselage acceleration is reduced by less than 0.10 m/s^2^, meeting the corresponding requirements of the Aircraft Landing Gear Strength Design Guide, which states that a landing gear should absorb and dissipate most of the aircraft landing impact energy within 0.8 s of landing, as far as possible to reduce the impact on the fuselage.

### 3.2. Model Verifications

There are many problems in verifying the accuracy of the model through experiments. The landing gear of the general aviation aircraft is not equipped with sensors, and mounting additional sensors on landing gear for flight experiments needs to meet the requirements of airworthiness. Also, flight requirements with faults are with high risks. In this section, the accuracy of the developed model is tested via comparing the simulated responses and practical data from airborne equipment during practical flights. The airborne equipment would offer acceleration of fuselage every second. RMS (root mean square) is chosen as an index to compare the vibration energy of fuselage during landing phase. Fuselage vibration is mainly decided by landing state (e.g., vertical landing speed), landing gear state (stiffness, damping, etc.) and runway state (e.g., runway irregularities), simulations in this paper aim at the dropping and grounding phase during landing, and landing gear should absorb and dissipate most of the aircraft landing impact energy within 0.8 s of landing. The comparisons in this section focus on the first second after wheel reach the ground. Both soft (0.451 m/s) and normal (0.969 m/s) landing conditions are chosen in this paper. According to the comparison results list in Table 2, the vibration energy of practical and simulated fuselage vertical acceleration signals is equivalent, with acceptable error.

## 4. LDGNet: A Deep Learning Model for Landing Gear Shock-Absorbing System Health Monitoring

### 4.1. Architecture of Structure

**Figure 3:**
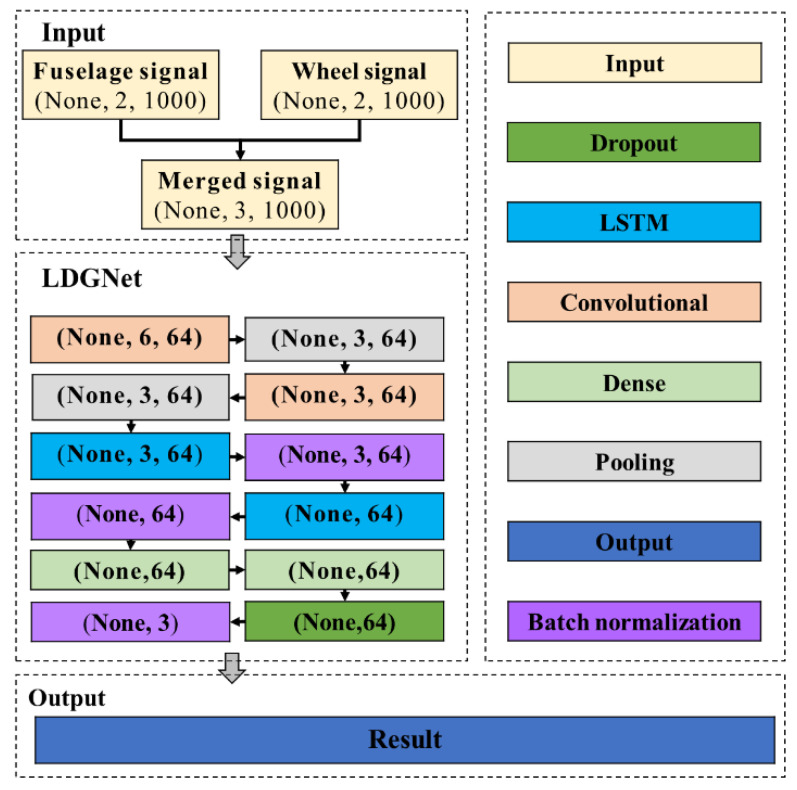
The architecture of LDGNet.

The structure of the designed LDGNet is shown in Figure 3. The sensor-acquired fuselage and wheel signal are merged as a 3 × 1000 matrix including time, fuselage and wheel signal when the wheel is in contact with ground during the aircraft landing phase. Firstly, the merged signal is imported to convolutional layers and pooling layers for feature extraction on spatial sequences. Features of the merged signal are extracted by two convolutional layers, each followed by a pooling layer that retains key features while reducing spatial dimensions through down-sampling, thereby decreasing computational complexity, controlling overfitting, and enhancing translation invariance. Secondly, the processed data matrix is imported to LSTM layers and batch normalization layers for feature extraction in time series, and is merged as 1D data for importing to dense layer. Softmax is applied in a dense layer for converting the original output of the network into a probability distribution, where each output value represents the predicted probability of the corresponding class, enabling the model to give a confidence level for each possible diagnosis. Dropout layer is designed to reduce the dependence on a specific combination of neurons, thereby effectively alleviating the overfitting problem, via randomly discarding a certain proportion of neuronal connections during the training process. The batch normalization layer is designed to effectively normalize the activation values into a fixed interval by calculating the mean and standard deviation on each small batch of data and making corresponding bias adjustments and scaling of the data. Compared with CNN and LSTM, the proposed LDGNet combines the advantages of CNN and LSTM, and the features of sensor-collected data in both spatial and time sequences are extracted to improve the performance of health monitoring.

### 4.2. Technique Route

**Figure 4:**
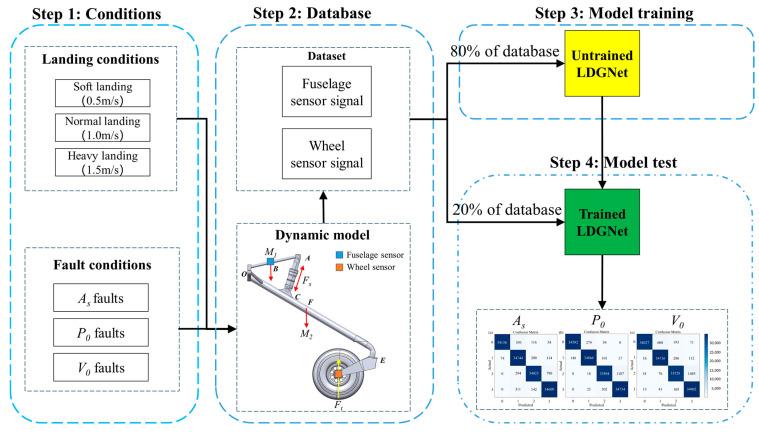
The technique route of LDGNet.

**Figure 5:**
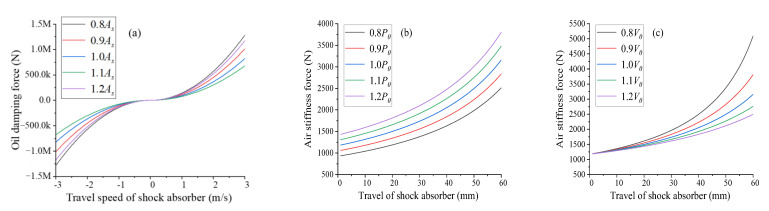
Fault conditions considered in this paper: (**a**) damping orifice area (*A_S_*) faults, (**b**) initial gas pressure (*P*_0_) faults and (**c**) initial gas volume (*V*_0_).

As shown in Figure 4, the technique route of this study mainly consists of the following four main steps:

Step 1: for training and testing the proposed health monitoring method, a database is built based on the developed nose landing model, including nose landing gear dynamic responses under various health states of shock absorbers and various landing conditions. Based on Section 3.1, the performance of an oleo-pneumatic shock absorber is mainly decided by the state of gas, oil and internal structure. The proposed fault database consists of common shock absorber faults, including initial gas pressure (*P*_0_) faults and initial gas volume (*V*_0_) faults which would cause variations in shock absorber stiffness, and damping orifice area (*A_S_*) faults which would cause variations in shock absorber damping. In the proposed shock absorber fault database, four different fault levels are considered for each fault type. As shown in Figure 5, faults considered in the shock absorber fault database would cause different influences on shock absorber parameters of the proposed landing gear model. Landing conditions mainly decide landing impact and have dramatic influences on dynamic responses of landing gear during landing. Landing conditions are considered as input for nose landing gear model. For ensuring the performance of the proposed health-monitoring method, a landing condition database is developed with common landing conditions during practical flights, including vertical landing speed of 0.5 m/s, 1.0 m/s and 1.5 m/s. The considered landing conditions are simulated via setting corresponding initial height of free fall for the proposed landing gear model.

Step 2: both fault conditions and landing conditions are sequentially applied to the developed dynamic model of nose landing gear developed in Section 3 for simulating dynamic responses under each condition. During each simulation, both vertical fuselage and wheel acceleration signal are acquired by the virtual fuselage and wheel sensor. Virtual sensor sampling frequence is 1000 Hz. All sensor-acquired signals are stored in the database for model training and testing.

Step 3: 80% of the database is used to train the proposed LDGNet. The training dataset, consisting of historical time-series records, is fed into the LDGNet to enable the model to learn the underlying patterns and dynamics associated with different fault conditions. The LDGNet architecture, which integrate CNN and LSTM, is optimized to capture both local features and long-term dependencies in the time-series data. During the training process, techniques such as batch normalization, dropout, and learning rate decay are applied to enhance the model’s robustness and prevent overfitting. The training phase continues iteratively until the model achieves convergence, ensuring that it can accurately classify various fault types based on the learned representations of the time-series data.

Step 4: after completing the training phase, the remaining 20% of the untrained time-series data are used to evaluate the performance of the proposed LDGNet. This testing dataset, which was not exposed to the model during training, serves as an independent benchmark to assess the model’s generalization capability and diagnostic accuracy. The untrained time-series data are fed into the LDGNet, and the model’s predictions are compared against the ground truth labels to generate a confusion matrix. The confusion matrix provides a comprehensive evaluation of the model’s performance, including key metrics such as accuracy, precision, recall and F1 score. These metrics offer insights into the model’s ability to correctly identify and classify different fault conditions, as well as its potential limitations in handling specific types of time-series patterns. The testing phase ensures that the LDGNet is not only effective on the training data, but also reliable and accurate when applied to new, unseen time-series data, making it a robust tool for real-world fault diagnosis applications.

## 5. Health Monitoring Results with LDGNet

**Figure 6:**
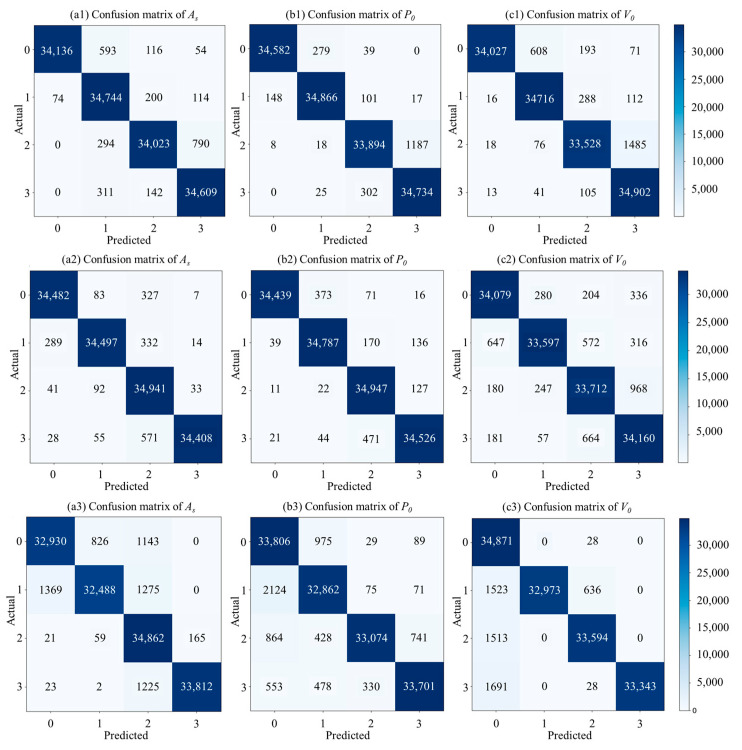
Confusion matrix of LDGNet under the landing speed of 0.5 m/s (**a1**–**c1**), 1.0 m/s (**a2**–**c2**) and 1.5 m/s (**a3**–**c3**).

After following the technique route, the confusion matrices are derived all considered conditions. The LDGNet running in Python 3.10 is programmed on a device with a 12th Gen Intel Core i5-12400KF CPU processor (Intel, Santa Clara, CA, USA) and an NVIDIA GeForce RTX 4060 GPU graphic processing unit (NVIDIA, Santa Clara, CA, USA). The confusion matrices under all considered conditions are shown in Figure 6, including all fault types under all considered landing conditions. In Figure 6, labels 0, 1, 2 and 3 refer to −20%, −10%, 10% and 20% of the normal parameter, respectively.

Based the confusion matrices shown in Figure 6, two indices are considered in this section to evaluate the performance of the proposed LDGNet in various aspects, which are accuracy and F1 score. As shown in Equation (4), accuracy is applied to evaluate the ability of judge the whole sample correctly by calculating the proportion of correctly classified samples in total samples. As shown in Equation (5), F1 score is applied to evaluate the stability of LDGNet, via calculating a weighted average of precision and recall.

(4) $$Accuracy=\frac{TP+TN}{TP+TN+FP+FN}\times 100\%$$

(5) $$\left\{\begin{array}{l} Precssion=\frac{TP}{TP+FP}\times 100\%\\ Recall=\frac{TP}{TP+FN}\times 100\%\\ F1=\frac{2\times (Precssion\times Recall)}{Precssion+Recall}\times 100\% \end{array}\right.$$

where *T* and *F* mean detection result of true and false correspondingly, and *P* and *N* mean positive and negative of the detection result.

### 5.1. Damping Orifice Area (A_S_) Fault Detection

**Figure 7:**
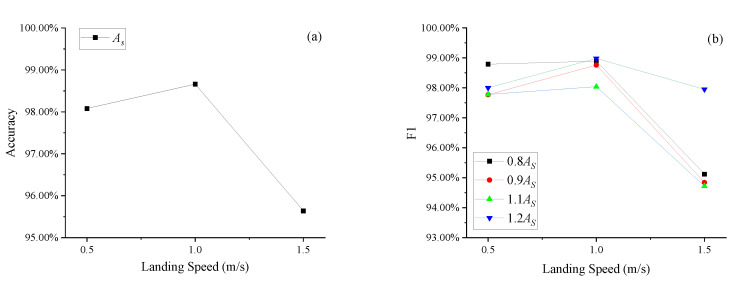
Accuracy (**a**) and F1 (**b**) of damping orifice area (*A_S_*) fault detection.

Figure 7 shows the accuracy (a) and F1 (b) of LDGNet for damping orifice area (*A_S_*) fault detection. As shown in Figure 7a, accuracies under normal (1.0 m/s) landing condition are greater than accuracy under soft (0.5 m/s) and heavy (1.5 m/s) landing condition. Accuracies under all landing speed conditions are larger than 95.00%, and accuracies under soft (0.5 m/s) and normal (1.0 m/s) are greater than 98.00%. As shown in Figure 7b, F1 scores have similar variation tendency with accuracies under damping orifice area (*A_S_*) conditions, F1 scores under normal landing condition are greater than F1 scores under soft and heavy landing condition. F1 scores under all landing speed conditions are all lager than 94.00%, and accuracy under soft and normal conditions are greater than 97.50%.

### 5.2. Initial Gas Pressure (P_0_) Fault Detection

**Figure 8:**
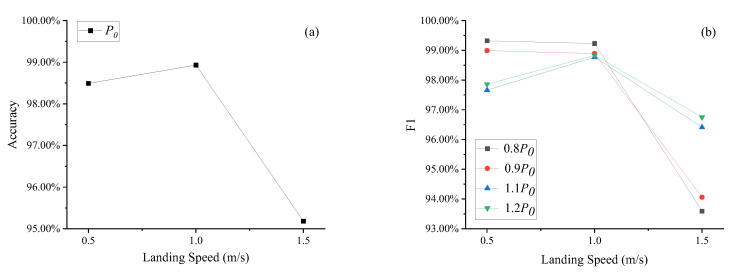
Accuracy (**a**) and F1 (**b**) of initial gas pressure (*P*_0_) fault detection.

Figure 8 shows the accuracy (a) and F1 score (b) of LDGNet for initial gas pressure (*P*_0_) fault detection. Similar with damping orifice area (*A_S_*) fault conditions, accuracies under normal (1.0 m/s) landing conditions are greater than accuracies under soft (0.5 m/s) and heavy (1.5 m/s) landing conditions. Accuracies under all landing speed conditions are all larger than 95.00%, and accuracies under soft (0.5 m/s) and normal (1.0 m/s) are greater than 98.00%. As shown in Figure 8b, F1 scores under all landing speed conditions are all larger than 93.00%, and accuracy under soft and normal landing conditions are greater than 97.50%. For *P*_0_ reduction conditions (0.8*P*_0_ and 0.9*P*_0_), F1 scores under normal landing condition are close to F1 scores under soft landing condition, and greater than F1 scores under heavy landing conditions. For *P_0_* increasing conditions (1.1*P*_0_ and 1.2*P*_0_), F1 scores under normal landing condition are greater than F1 scores under both soft and heavy landing conditions.

### 5.3. Initial Gas Volume (V_0_) Fault Detection

**Figure 9:**
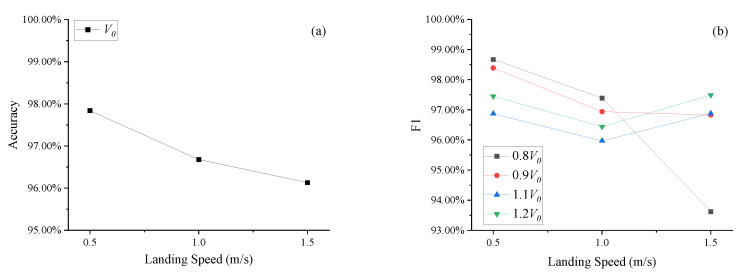
Accuracy (**a**) and F1 (**b**) of initial gas volume (*V*_0_) fault detection.

Figure 9 shows accuracy (a) and F1 (b) of LDGNet for initial volume (*V*_0_) fault detection. As shown in Figure 9a, accuracies reduce with an increase in landing speed. Accuracies under all landing speed conditions are greater than 95.00%. As shown in Figure 9b, F1 scores under all conditions are greater than 93%, and F1 scores under soft and normal landing conditions are greater than 95.50%. For *V*_0_ reduction conditions (0.8*V*_0_ and 0.9*V*_0_), F1 scores reduce with an increase in landing speed. For *V*_0_ increase conditions (1.1*V*_0_ and 1.2*V*_0_), F1 scores under normal landing condition are greater than F1 scores under soft and heavy landing conditions.

In summary, the developed LDGNet is proved with its accuracy for all considered fault conditions. Based on the analyses, it is found that the accuracy of LDGNet under soft and normal landing conditions is better than heavy landing conditions, which is due to the impact during heavy landing would domain the dynamic responses of landing and would reduce the influence of shock absorber states on landing gear.

### 5.4. Comparison

**Figure 10:**
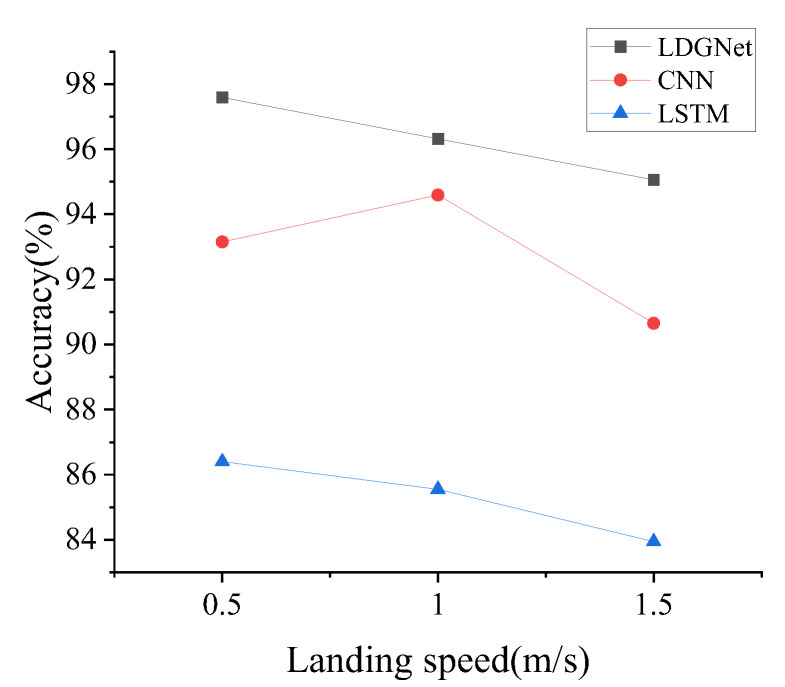
Comparison between LDGNet, CNN and LSTM.

This subsection focuses on comparison between the proposed LDGNet and existing methods, including the CNN and LSTM. The database built in Section 4.1 is applied to the CNN and LSTM for model training and accuracy verification. The accuracy of under all landing conditions are compared to evaluate the performance of the proposed LDGNet. As shown in Figure 10, the accuracy of LDGNet is higher than CNN and LSTM under all landing conditions. Via extracting features in the time and spatial domain, the developed LDGNet could offer a better performance in health monitoring than CNN and LSTM.

## 6. Conclusions

Faults occurring in the landing gear shock-absorbing system would have negative effects on passenger comfort and aircraft safety. This paper aims at exploring an on-board health monitoring method of landing gear shock-absorbing systems with a simple sensor network, which is easier to pass airworthiness. The work in this paper is concluded as follows:

(1) This paper first discusses the difficulties of developing an on-board health monitoring method for a landing gear shock-absorbing system. A simple sensor network, consisting only of fuselage and wheel accelerometers, is designed to meet the requirements of airworthiness. A deep learning method is proposed to explore the health monitoring model, training the database from dynamic simulations.
(2) LDGNet is developed for conducting health monitoring for the landing gear shock-absorbing system. The LDGNet consists of 12 different layers including convolutional layers, pooling layers, LSTM layers, etc., and it only requires vertical fuselage and wheel acceleration as input.
(3) For building the training database for the proposed LDGNet, a nose landing gear dynamic model is developed for a certain general aviation aircraft. The model is verified by comparing simulation results with data from practical flights. Virtual sensors are mounted on fuselage and wheel of the developed model for acquiring simulated dynamic responses under all considered conditions. Common typical fault conditions (*A_S_*, *P*_0_ and *V*_0_) and landing conditions (soft, normal and heavy landing) are considered when conducting simulations for the training database.
(4) The proposed LDGNet is trained by the simulated database. Two indices are applied to evaluate the performance of LDGNet, including accuracy and F1 score. Based on analyses, the accuracies of all considered fault conditions are greater than 95.00% under all considered landing conditions, and the accuracies under soft and normal landing conditions are greater than accuracies under heavy landing conditions. F1 scores of all considered fault conditions are greater than 93.00% under all considered landing conditions. For soft and normal landing conditions, the F1 scores of damping orifice area (*A_s_*) faults, initial gas pressure (*P*_0_) faults and initial volume (*V*_0_) faults are greater than 97.50%, 97.50% and 95.50% correspondingly.

Future researches will focus on optimizing the database with more detailed landing gear models, which would consider the structure, flexibility and all landing gears in the aircraft. Field tests will be conducted to enrich the database from practical flights, and the proposed LDGNet will be optimized by the new database.

## Figures and Tables

**Table 1:** Joints and DOFs list for nose landing gear model, where *X*, *Y* and *Z* indicate the longitudinal, vertical and lateral motion; *α*, *β* and *γ* indicate the roll, yaw and pitch motion; √ and × are representing has and do not have that DOF.

Component 1	Component 2	Joint	DOF					
			*X*	*Y*	*Z*	*α*	*β*	*γ*
Ground	Fuselage	Moving	×	√	×	×	×	×
Fuselage	Engine Frame	Fixed	×	×	×	×	×	×
Engine Frame	Strut	Rotating	×	×	×	×	×	√
Strut	Fork	Rotating	×	×	×	×	√	×
Fork	Rim	Rotating	×	×	×	×	×	√
Rim	Tire	Fixed	×	×	×	×	×	×

**Table 2:** Comparisons of practical flight data and simulation data.

Practical Data		Simulated Data		Errors	
Landing Speed (m/s)	Vertical Acceleration (m/s^2^)	Landing Speed (m/s)	Vertical Acceleration (m/s^2^)	Speed	Acceleration
0.451	2.4525	0.5	2.7397	10.9%	11.7%
0.969	4.4145	0.969	4.2839	0.0%	2.98%

## Data Availability

Data will be made available on request.
